# Evaluation of dental morphometrics during the orthodontic treatment

**DOI:** 10.1186/1475-925X-13-68

**Published:** 2014-06-03

**Authors:** Magdaléna Kašparová, Aleš Procházka, Lucie Grajciarová, Mohammadreza Yadollahi, Oldřich Vyšata, Tat’jana Dostálová

**Affiliations:** 1 Department of Paediatric Stomatology, The Second Medical Faculty, Charles University, V Úvalu 84, 150 06 Prague 5, Czech Republic; 2 Department of Computing and Control Engineering, Institute of Chemical Technology in Prague, Technická 5, 166 28 Prague 6, Czech Republic; 3 Department of Neurology, Charles University, Sokolská 581, 500 05 Hradec Králové, Czech Republic

**Keywords:** Orthodontic modelling, Geometric morphometrics, Digital models, Dental arch features, Digital signal processing, Regression analysis, Computational intelligence

## Abstract

**Background:**

Diagnostic orthodontic and prosthetic procedures commence with an initial examination, during which a number of individual findings on occlusion or malocclusion are clarified. Nowadays we try to replace standard plaster casts by scanned objects and digital models.

**Method:**

Geometrically calibrated images aid in the comparison of several different steps of the treatment and show the variation of selected features belonging to individual biomedical objects. The methods used are based on geometric morphometrics, making a new approach to the evaluation of the variability of features. The study presents two different methods of measurement and shows their accuracy and reliability.

**Results:**

The experimental part of the present paper is devoted to the analysis of the dental arch objects of 24 patients before and after the treatment using the distances between the canines and premolars as the features important for diagnostic purposes. Our work proved the advantage of measuring digitalized orthodontic models over manual measuring of plaster casts, with statistically significant results and accuracy sufficient for dental practice.

**Conclusion:**

A new method of computer imaging and measurements of a dental stone cast provides information with the precision required for orthodontic treatment. The results obtained point to the reduction in the variance of the distances between the premolars and canines during the treatment, with a regression coefficient *RC*=0.7 and confidence intervals close enough for dental practice. The ratio of these distances pointed to the nearly constant value of this measure close to 0.84 for the given set of 24 individuals.

## Introduction

It is becoming increasingly evident in orthodontics and dentofacial orthopedics that the timing of the onset of treatment may be as critical as the selection of a specific treatment protocol. By beginning at the patient’s optimal maturational stage, the most favorable response with the lowest potential morbidity can be anticipated. The issue of optimal timing for dentofacial orthopedics is closely linked to the identification of growth periods that can contribute significantly to the correction of skeletal imbalances in the individual patient. Cephalometric investigations on longitudinal samples have identified a pubertal spurt in mandibular growth that possesses wide individual variations in onset, duration, and rate [1-4].

The multidisciplinary dental care of patients is not a simple matter. New prosthodontic methods, including implant insertion, can be instituted using therapy by the surgeon, orthodontist and prosthodontist [5]. Dental casts play an important role in the diagnosis and treatment planning [6-8] in prosthodontics and orthodontics. Digitalization [9,10] is an important part of medicine using paperless patient information systems as virtual charts, digital photographs, and digital dental cross.

Although plaster casts are the gold standard in treatment planning, not only in orthodontics, the replacement of plaster dental casts by their digital models [11-14] can be advantageous, especially in saving space in storage areas, the efficiency of having patient records accessible through a computer, the possibility of sharing the models with other specialists needed during the therapy, and the possibility of accurate measurements and the use of diagnosis setups.

The analysis of a study cast, consisting of the three-dimensional assessment of the maxillary and mandibular dental arches and the maximal intercuspal relationship, is one of the basic tools of diagnosis and treatment planning. The arch form, dimensions, and variations obtained from orthodontic and prosthodontic treatment have been the subject of study for many years now [8]. Some authors have tried to identify a geometric curve that would facilitate the accurate definition of arch forms [15]. The first studies showing the importance of 3D dental arch analysis were conducted in the previous century. Bonwill and Hawley described the alignment of the upper anterior teeth as a circumference arch; whilst MacConaill and Scher maintained that the dental arch resembled a catenary curve [16,17]. Izard, in trying to relate the dimension of the dental arch to the facial dimensions, found that the arch form could be accurately represented by an elliptical curve [18], as can be also found in our group of plaster casts.

Both methods, manual measurement and measurement using 3D models of dental casts [19-23], have advantages and disadvantages which can influence the result of the measurement and therefore also the treatment planning, treatment evaluation or face reconstruction [24].

**Figure 1:**
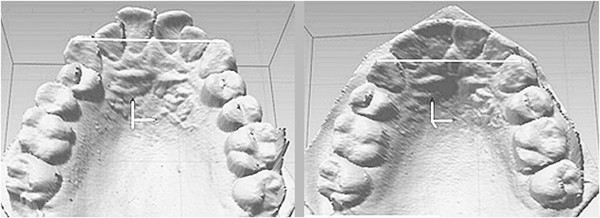
Orthodontic measurement of the distance 3-3 using a digital picture of a plaster cast before and after treatment.

Geometric morphometrics [25-31] represents a new approach to the evaluation of variability, and not only in medicine (Figure 1). Its methods are based mainly on the 3D co-ordinates of homologous landmarks that describe the studied object. The co-ordinates thus represent a complete set of geometric information related to the object under study [32]. This system enables the differentiation of variability by both size and shape. The quantification of shape and size specifies and renders more accurate results than those that have been obtained to date with other methods, thus increasing the reliability and accuracy [33,34] of dental geometry measurements in clinical practice.

The aim of this study is to validate the accuracy and use of digitized dental plaster casts in dental practice [35,36], using a group of 48 plaster casts. It forms a contribution to the study of dental arch parameters using segmentation, classification and registration [37,38] of orthodontic data.

## Methods

### Data acquisition

We created a set of 24 patient’s plaster casts, randomly selected from the patients who underwent an orthodontic treatment at the Department of Orthodontics, 2nd Medical Faculty, Charles University, Prague, Czech Republic. Informed consents were obtained from all patients or their legal representatives. No ethical approval was required for this study.

All patients suffered from Class II malocclusion and had already finished orthodontic treatment by a fixed orthodontic appliance and extraction of the first premolars. There were no orthodontic appliances present. Two study plaster casts of the upper jaw (pre- and post treatment) were made of each patient, which means there were 48 plaster casts. All of the plaster casts were of high quality with no fractures or other damage.

A Roland LPX-250 scanner was used to scan all of the plaster casts. This is non-contact laser scanner which picks up several points to create coordinates and converts them into 3D data [39,40] with a lateral resolution of 200 *μ*m. Multiple scanning can be done in up to six planes. The laser automatically works in conjunction with the Dr. Picza program, in which scanned data is entered and from which rough models are exported to other programs. The subsequent adjustment of the models involves the projection of surface re-scans onto the individual rotational scans, adjustment of all scans and re-scans (e.g. identification and removal of abnormal surfaces, or deletion of excessively long surfaces), and the registration and merging of individual re-scans with the rotational scan. The imperfect surface is further adjusted using a smoothing function; the small apertures that develop during adjustment of the scans are filled in according to the original model. The resulting models may then be further analysed using existing software, i.e., sections may be made according to previously defined planes, and the shape of any structure may be measured.

The Pixform software package was used for measuring the digitalized plaster casts. All models were displayed in the same colour and zoom and rotation functions were used to find the ideal point of measurement.

For the purposes of this study, the measurements of the dental arch width were made twice on each of the casts and twice on each of the digital models of dental casts. All measurements were made by a single examiner in the same conditions and were repeated in 72 hours.

In the first part of the study, the measurements of the width of the dental arch between the canines (3-3) and the second premolars (5-5) of the 24 sets of plaster casts were made manually. A sliding scale was used for this purpose. While measuring the plaster casts, the casts were held in the hand and manual rotation was used to find the tips in the cusps of the measured teeth.

In the second part of the study, the same measurement as in the first part was made on digitalized plaster casts of the study group. The casts were scanned on a rotating plate from a position perpendicular to the occlusal plane. The raw scan data were processed using Pixform reverse engineering software (Roland DG). This procedure included cleaning, merging of multiple scans, hole-filling, decimating, smoothing, and global remeshing.

The measured values used for statistical analysis are presented in Table 1. The set of 24 patients had been tested before and after the orthodontic treatment using plaster casts for all patients. The distances between the reference points [41] associated with the canines (3-3) and the premolars (5-5) have been measured (twice) both manually and digitally using the 3D model.

**Table 1:** Distances [mm] between corresponding canines (3-3) and premolars (5-5) measured twice both for digital models and plaster casts for 24 patients (i) before and (ii) after the treatment

	***Digital model measurement***					***Plaster cast measurement***			
**Set**	***1***		***2***			***1***		***2***	
	**3-3**	**5-5**	**3-3**	**5-5**		**3-3**	**5-5**	**3-3**	**5-5**
1 (i)	29.98	39.96	28.78	40.03		28.2	40.1	28.6	40.3
(ii)	30.14	37.58	31.14	37.77		29.7	37.6	29.6	38.0
2 (i)	36.63	43.46	36.75	42.81		35.0	42.7	36.2	42.5
(ii)	34.57	40.73	33.77	40.20		33.3	41.9	33.9	41.2
3 (i)	33.44	44.23	33.54	43.49		32.5	44.8	32.6	44.8
(ii)	32.52	40.15	32.62	38.25		31.8	39.5	32.3	39.8
4 (i)	33.38	40.60	33.90	39.96		31.9	40.4	32.4	40.0
(ii)	33.37	39.56	34.06	40.04		32.6	40.6	33.3	41.8
5 (i)	36.02	46.86	36.12	46.95		35.4	47.1	35.2	47.0
(ii)	33.38	40.67	33.72	40.18		32.8	40.8	33.3	40.9
6 (i)	29.60	42.77	29.72	42.15		29.0	43.1	29.9	43.5
(ii)	32.04	42.61	31.80	41.91		31.2	42.8	31.8	42.8
7 (i)	37.85	47.17	38.31	47.58		37.1	46.8	37.4	47.9
(ii)	36.24	42.46	36.58	41.61		36.1	47.8	35.4	42.4
8 (i)	34.26	41.06	33.79	41.11		33.1	41.8	33.8	41.3
(ii)	33.11	40.65	33.96	39.50		32.9	40.5	33.1	40.8
9 (i)	37.38	42.02	37.86	41.11		36.7	42.4	36.0	42.1
(ii)	34.26	39.42	33.96	39.50		34.1	39.8	34.1	40.2
10 (i)	37.53	44.16	37.74	44.42		34.7	43.9	33.9	44.0
(ii)	37.13	42.69	37.70	42.16		37.3	41.9	37.6	42.1
11 (i)	36.44	45.11	36.29	44.97		35.9	43.8	36.0	44.1
(ii)	35.50	42.53	36.01	42.41		35.5	44.9	35.9	44.8
12 (i)	31.19	38.52	30.18	39.21		31.2	39.0	32.9	39.9
(ii)	33.48	40.70	34.12	40.39		32.4	39.4	33.0	39.7
13 (i)	28.53	42.92	28.67	42.81		28.1	42.9	28.5	42.8
(ii)	33.25	40.68	32.59	41.27		33.4	40.4	32.8	40.0
14 (i)	37.76	46.42	37.41	46.93		36.8	46.9	36.7	45.5
(ii)	37.84	44.74	37.62	44.72		37.6	44.0	37.6	43.8
15 (i)	37.87	42.65	38.08	42.94		37.5	42.0	37.2	41.9
(ii)	37.72	42.25	37.86	41.62		37.9	42.2	37.8	42.0
16 (i)	33.28	37.88	32.86	37.78		32.6	37.8	32.1	38.1
(ii)	33.30	41.04	33.33	40.37		33.9	41.5	32.8	41.2
17 (i)	33.93	41.86	33.61	41.70		33.0	41.5	33.1	42.0
(ii)	33.71	41.08	33.52	41.24		33.1	40.8	32.6	41.8
18 (i)	35.77	43.61	36.56	43.56		35.4	43.4	35.0	43.6
(ii)	36.07	41.89	35.81	42.91		35.8	41.4	35.3	41.9
19 (i)	28.84	41.28	29.44	41.02		28.9	41.1	29.1	41.4
(ii)	33.64	37.90	33.49	38.08		33.0	38.9	32.6	38.4
20 (i)	30.67	42.75	29.99	42.64		30.0	42.9	30.3	42.4
(ii)	34.95	41.88	34.51	42.26		34.2	41.2	33.8	41.9
21 (i)	35.17	40.89	35.73	41.24		34.6	41.0	34.0	41.1
(ii)	35.25	41.27	35.62	40.52		34.3	41.4	34.9	40.7
22 (i)	42.58	47.39	43.06	48.19		41.6	47.9	40.6	47.8
(ii)	39.22	45.14	39.31	45.82		39.0	45.3	39.1	45.7
23 (i)	34.51	40.47	34.81	40.86		34.4	40.6	33.7	41.0
(ii)	35.13	41.96	34.46	41.17		34.3	40.8	34.8	41.1
24 (i)	29.64	33.26	29.39	32.72		29.1	33.1	29.1	32.9
(ii)	32.30	39.20	31.88	39.20		31.4	38.9	31.2	39.0

### Data processing

That the measured distances obey a Gaussian normal distribution *N*(*μ*,*σ*^2^) is the main assumption of statistical testing [42-46]. The evaluation of errors in sequences ${\left\{d(n)\right\}}_{n=1}^{N}$ standing for differences in repeated measurements resulting either from the computational digital model or from the manual measurement on the plaster cast was based upon estimation of their mean values and standard deviations

(1) $$\bar{d}=\frac{1}{N}\overset{N}{\underset{i=1}{\sum }}d(i)\;\text{and}\;s=\sqrt{\frac{1}{N-1}\overset{N}{\underset{i=1}{\sum }}{\left(d(i)-\bar{d}\right)}^{2}}$$

before and after the treatment using different kinds of measurements with the confidence intervals

(2) $$\begin{array}{lcr} \mu & \in & \langle \bar{d}-t_{N-1,\frac{\alpha }{2}}\;\frac{s}{\sqrt{N}},\;\bar{d}+t_{N-1,\frac{\alpha }{2}}\;\frac{s}{\sqrt{N}}\rangle \end{array}$$

(3) $$\begin{array}{lcr} \sigma^{2} & \in & \langle \frac{(N-1)\;s^{2}}{\chi_{N-1,\frac{\alpha }{2}}^{2}},\;\frac{(N-1)\;s^{2}}{\chi_{N-1,1-\frac{\alpha }{2}}^{2}}\rangle \end{array}$$

for the *t* and *χ*^2^ distributions, respectively, and selected confidence level *p*=100 (1−*α*)%.

The comparison of the results obtained from the computational digital models and from the plaster casts [47] was studied through histograms and the distribution of the measured values. The differences in the distances obtained from the digital models and those from the plaster casts were tested by the pair *t*-test. The same test was used for the differences in the distances before and after the treatment. The precisions of the distance measurement of the digital models and the plaster casts were tested using the standard *F*-test [48] for the null hypothesis that the two normal sets have the same variance.

The correspondence of the distances between the canines 3-3 and the premolars 5-5 formed sequences ${\left\{x(n)\right\}}_{n=1}^{N}$ and ${\left\{y(n)\right\}}_{n=1}^{N}$ for the *N* selected individuals. The correlation coefficient *r*_*x* *y*_ between these two sequences was calculated through the relation

(4) $$r_{\text{xy}}=\frac{C_{\text{xy}}}{\sqrt{C_{\text{xx}}\;C_{\text{yy}}}}$$

where the cross-covariance is given by

(5) $$C_{\text{xy}}=\frac{1}{N}\overset{N}{\underset{i=1}{\sum }}(x(i)-\bar{x})(y(i)-\bar{y})$$

and the mean by

(6) $$\bar{x}=\frac{1}{N}\overset{N}{\underset{i=1}{\sum }}x(i)\;\text{and}\;\bar{y}=\frac{1}{N}\overset{N}{\underset{i=1}{\sum }}y(i).$$

All the tests were performed at the selected significance level *α*=0.05 separately for the distances measured between the canines (3-3) and those between the premolars (5-5). Selected statistical tests were performed in the Matlab Statistical Toolbox [49,50].

## Results

The plaster casts were evaluated using classical methods and geometrical morphometrics. Changes in the transverse direction of growth were determined from the distance between the tips of the permanent canines and the premolars, which we denote by 3-3 and 5-5.

**Figure 2:**
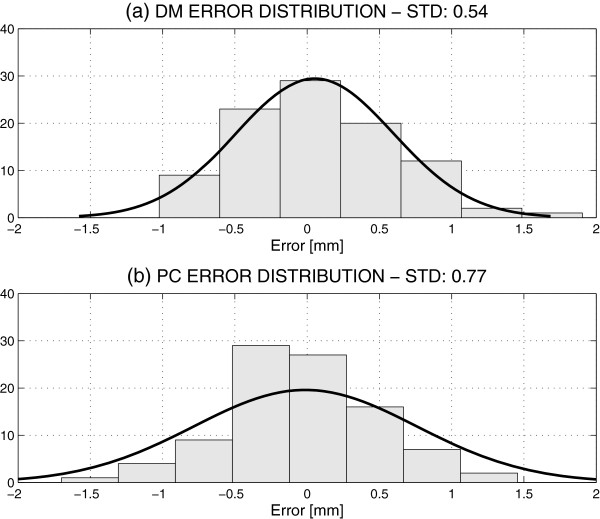
Comparison of distributions of errors of successive measurements of distances between corresponding teeth using (a) the digital model (DM) and (b) the plaster cast (PC).

The distance measurements acquired from the digital models and the plaster casts had similar precisions [51-53]. The mean value of the differences between these measurements was close to zero both for digital and manual measurements, and had similar standard deviations, as seen in Table 2. Figure 2 presents the distribution of 96 errors resulting from 192 observations both for digital and manual measurements.

**Table 2:** Statistical characteristics including confidence intervals (CI) of digital model (DM) and plaster cast (PC) before and after the treatment

***Characteristics***	***Mean***	***95% CI***	***Std***	***95% CI***
	***[mm]***		***[mm]***	
DM measurement differences	0.05	〈−0.06;0.16〉	0.54	〈0.48;0.63〉
PC measurement differences	-0.01	〈−0.17;0.14〉	0.77	〈0.68;0.89〉
DM 3-3 before the treatment	34.27	〈32.71;35.82〉	3.68	〈2.86;5.16〉
PC 3-3 before the treatment	33.48	〈32.09;34.87〉	3.30	〈2.56;4.63〉
DM 5-5 before the treatment	42.36	〈40.97;43.75〉	3.29	〈2.56;4.61〉
PC 5-5 before the treatment	42.39	〈41.04;43.75〉	3.21	〈2.49;4.50〉
DM 3-3 after the treatment	34.53	〈33.65;35.41〉	2.09	〈1.63;2.93〉
PC 3-3 after the treatment	34.09	〈33.13;35.05〉	2.28	〈1.77;3.19〉
DM 5-5 after the treatment	41.08	〈40.31;41.85〉	1.83	〈1.42;2.57〉
PC 5-5 after the treatment	41.38	〈40.55;42.21〉	1.96	〈1.53;2.76〉

Digital measurements provide slightly better results, with a smaller standard deviation. The distances between the canines (3-3) and the premolars (5-5) before and after the treatment obtained by digital and manual measurements are presented in Table 2 together with their 95% confidence intervals (CI) as well. These results correspond to registering digital models obtained after and before the treatment [38].

The *t*-test used for comparison of the distances measured (i) on digital models and (ii) plaster casts proved that data in both vectors are random samples from normal distributions with equal means and variances at the 5% significance levels with the 95% confidence interval of the difference between the population means for distances 3-3 in the range 〈−0.55;1.78〉, and for distances 5-5 in the range 〈−1.25;0.92〉. The *F*-test applied to the two different kinds of measurements proved that the null hypothesis of the variances’ being equal can be accepted at the significance level of 5% with value *p*=0.58.

**Figure 3:**
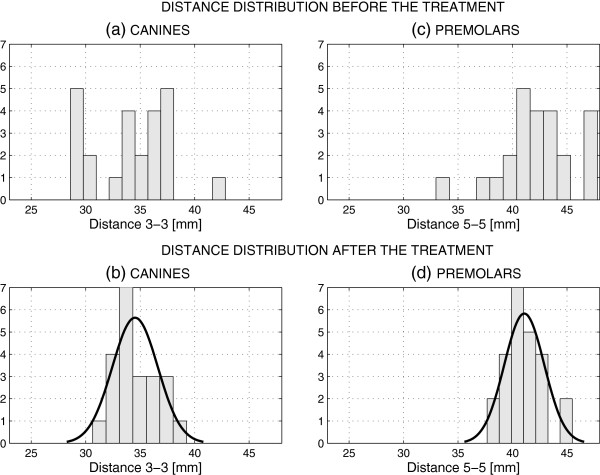
Distribution of distances between selected teeth before and after the treatment presenting (a) initial distribution for canines (3-3), (b) final distribution for canines (3-3), (c) initial distribution for premolars (5-5), (d) final distribution for premolars (5-5) defining their position changes.

The treatment results in changes in the differences between corresponding teeth. Figure 3 presents the distribution of these changes resulting from digital measurements for 24 individuals summarized in Table 1. While the distribution of the differences before the treatment covers a wide range of values, both for canines and premolars, this distribution is close to the normal distribution after the treatment.

**Figure 4:**
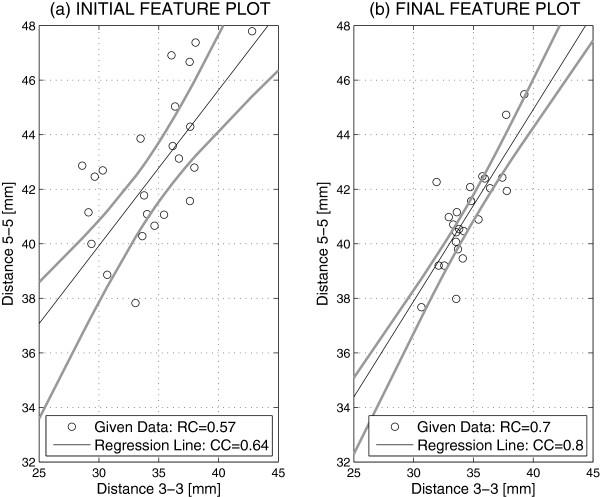
Regression of dental arch features defined by distance between canines (3-3) and premolars (5-5) (a) before and (b) after the treatment using digital dental models with regression coefficients (RC), correlation coefficients (CC) and 95% confidence intervals.

Figure 4 presents the correlation of the distances between the canines (3-3) and the premolars (5-5) before and after the treatment for 24 individuals summarized in Table 1. Assuming a linear regression it was possible to evaluate the correlation coefficient that grew from its value *RC*=0.64 before the treatment to *RC*=0.8 after the treatment with feature pair positions close to the regression line. The regression coefficient obtained for the given set of individuals after the treatment had a value of *CC*=0.70 defining the typical ratio of the distances between the premolars (5-5) and the canines (3-3) for the given set of individuals. Figure 4 presents 95% confidence intervals of correlation coefficients value as well.

**Figure 5:**
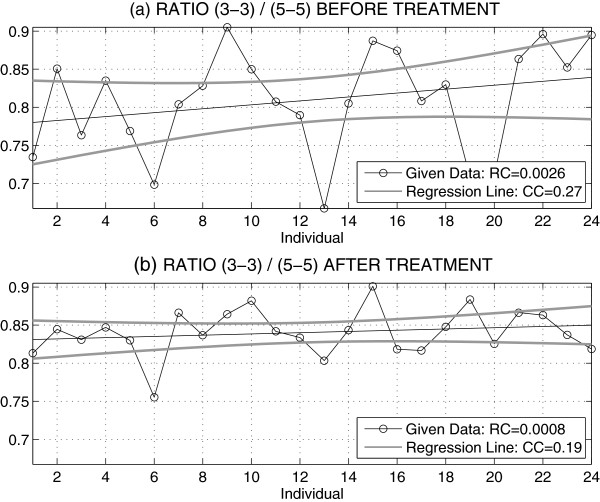
Ratio of distances between canines (3-3) and premolars (5-5) (a) before and (b) after the treatment with regression coefficients (RC), correlation coefficients (CC) and 95 % confidence intervals.

**Figure 6:**
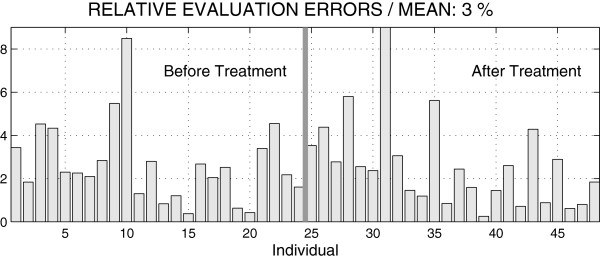
Relative differences of the ratio of distances between canines (3-3) and premolars (5-5) evaluated from digital models and plaster casts.

The affect of the orthodontic treatment to the ratio of distances between the canines (3-3) and the premolars (5-5) before and after the treatment for 24 individuals is presented in Figure 5. The regression coefficient value decreased from the value *RC*=0.0026 to *RC*=0.0008 pointing to nearly constant value of this ratio with its mean 0.84 and standard deviation 0.03 for patients after the treatment.Figure 6 presents the comparison of measures evaluated from digital models and plaster casts. The ratio of distances between canines (3-3) and premolars (5-5) of all observations has its mean relative difference of 3 % between digital and manual measurements.

## Discussion

With present day knowledge, the analysis of study casts as a whole is often considered to have limited diagnostic value [54]. A new method of computer imaging and measurements on a dental stone cast is a ubiquitous tool in dentistry and aids in the recording of the precise information [55,56] required for treatment, does not destroy hard dental tissues, and provides stable results. The present paper monitored this process, step-by-step. In both methods we have to count on the possibility of subjective mistakes, which means both methods are influenced by the skills of the measuring person. Because of this fact, we can not reliably say which of the two methods mentioned above is better.

The statistical analysis of the geometric measurements obtained from the digital models points to a decrease in the variances of the distances between the premolars (5-5) related to the distances between the canines (3-3) after the treatment, and to the regression coefficient being close to 0.7 for the given set of individuals, with a high correlation and close confidence intervals. The mean value of ratio of distances between the canines (3-3) and the premolars (5-5) after the treatment for 24 individuals is 0.84 with the regression coefficient *RC*=0.0008 close to zero. This result corresponds with typical dental arch parameters.

## Conclusion

This contribution compared the accuracy of two different methods used for the measurement of distances in orthodontics and used for treatment planning and its evaluation. The resulting digital model was then used for the analysis of selected dental morphometrics.

As a result of our measurements, we can say that measurements of digital models have the same accuracy as measurements of plaster casts, and they moreover allow more convenient way of data processing in clinical practice.

This conclusion is in accordance with modern trends to move to digital formats for orthodontic models, as suggested by the American Board of Orthodontics [57-60]. Physical casts, either plaster casts or models printed by 3D digital printers, can be replaced by digital models in many cases.

A specific following study devoted to distances between the canines (3-3) and the premolars (5-5) before and after the treatment for 24 individuals performed by digital models pointed to the nearly constant value of their ratio close to 0.84. The mean value of the relative difference between values evaluated from the digital model and plaster cast was 3%.

Our further research will be devoted to more precise 3-D modelling and to an algorithmic approach to the evaluation of the orthodontic parameters used for the treatment and follow up care of patients. The digital detection of reference points on three dimensional models and the volume registration [61] of models in different stages of the treatment will be studied as well.

## Competing interests

The authors declare that they have no conflict of interests.

## Authors’ contributions

The paper presents results of the close interdisciplinary collaboration of two research groups. Authors from the Department of Paediatric Stomatology of the Second Medical Faculty of Charles University were responsible for data acquisition resulting from their own medical treatment and for correct interpretation of results. Authors from the Department of Computing and Control Engineering of the Institute of Chemical Technology and from the Neurological Department of Charles University belonging to the Digital Signal and Image Processing Research Group were responsible for mathematical analysis of biomedical data and their statistical evaluation. All authors read and approved the final manuscript.
